# Association of triglyceride-glucose index with helicobacter pylori infection and mortality among the US population

**DOI:** 10.1186/s13098-024-01422-9

**Published:** 2024-08-01

**Authors:** Xing-Yun Zhu, Yu-Jun Xiong, Xiang-Da Meng, Hua-Zhao Xu, Lili Huo, Wei Deng

**Affiliations:** 1 Department of Endocrinology, Beijing Jishuitan Hospital, Capital Medical University, No. 31, East Xinjiekou Street, Xicheng District, Beijing, 100035 People’s Republic of China; 2 Department of Gastroenterology, Beijing Hospital, National Center of Gerontology, Institute of Geriatric Medicine, Chinese Academy of Medical Sciences, Beijing, 100370 P.R. China; 3 Department of Hernia and Abdominal Wall Surgery, Peking University People’s Hospital, Beijing, 100044 China; 4 Hospital Administration Office, Beijing Hospital, National Center of Gerontology, Institute of Geriatric Medicine, Chinese Academy of Medical Sciences, Beijing, China

**Keywords:** H. Pylori infection, TyG index, NHANES, Mortality

## Abstract

**Background:**

Limited research has explored the potential association between the Triglyceride-Glucose (TyG) and mortality, especially in individuals with Helicobacter pylori (H. pylori) infection. This study seeks to investigate the correlation between the TyG index and H. pylori infection and investigate whether the associations between the TyG index exposure and all-cause mortality are mediated by H. pylori infection.

**Methods:**

The study utilized data from the National Health and Nutrition Examination Survey (NHANES) 1999–2018, incorporating a final sample size of 2,187 participants. Both univariable and multivariable-adjusted logistic regression analyses were employed to examine the relationship between H. pylori infection and relevant covariates. To assess the association between TyG index, and all-cause mortality in individuals with or without H. pylori infection, Cox regression analysis, and restricted regression cubic spline analysis were implemented.

**Results:**

A significant positive correlation was observed between the TyG index and an elevated risk of H. pylori infection [OR 1.157, 95% CI (1.383 ~ 1.664)]. This correlation persisted even after adjusting for confounding factors [OR 1.189, 95% CI (1.003, 1.411), *P* < 0.05]. Furthermore, in patients with positive H. pylori infection, a noteworthy nonlinear correlation between the TyG index and all-cause mortality was identified (*P* = 0.0361). With an increase in the TyG index, all-cause mortality exhibited a corresponding rise, particularly following adjustment for all potential confounding factors. Conversely, in patients with negative H. pylori infection, no significant association was observed between the TyG index and all-cause mortality after adjusting for potential confounding factors.

**Conclusion:**

A higher TyG index was linked to increased H. pylori infection risks. Participants in the higher quantile group of the TyG index are positively associated with higher all-cause mortality compared to the higher quantile group of the TyG index in H. pylori-positive participants instead of H. pylori-negative participants.

**Supplementary Information:**

The online version contains supplementary material available at 10.1186/s13098-024-01422-9.

## Introduction

Helicobacter pylori (H. pylori) infection, a prevalent colonization of the gastric mucosa, infects over 4.4 billion people worldwide and remains a significant etiological factor in various gastrointestinal disorders [1, 2]. Extensive research has been dedicated to unraveling the mechanistic intricacies linking H. pylori to oxidative stress, characterized by an imbalance between reactive oxygen species (ROS) production and antioxidative defense mechanisms [3]. In a study conducted by Xiong et al., dietary inflammation index was positively correlated with mortality, particularly in individuals with H. pylori infections [4]. Hence, activation of inflammatory cascades and perturbations in cellular redox homeostasis are recognized as pivotal contributors to the pathogenesis of H. pylori-associated diseases, including gastritis, peptic ulcers, and gastric malignancies [3, 5, 6].

The ROS induced by H. pylori infection may disrupt metabolic processes, contributing to various metabolic disorders such as diabetes and dyslipidemia. Prior research also suggests that H. pylori infection could elevate diabetes risk by up to 27% [7]. Additionally, it is identified as a significant independent risk factor for dyslipidemia, beyond other cardiovascular risk factors [8, 9]. The Triglyceride-Glucose (TyG) index, calculated from fasting plasma glucose (FPG) and fasting triglyceride (TG) levels, serves as an indicator of metabolic dysfunction. This index, integrating the dual impacts of blood glucose and lipid levels, is regarded as a more convenient, accurate, and reliable method for assessing the risk of insulin resistance and cardiovascular diseases [10–12]. Furthermore, the TyG index has been associated with all-cause mortality and cardiovascular mortality [13]. Based on existing studies, we hypothesize that H. pylori infection could influence the TyG index, thereby impacting all-cause mortality. However, the interrelation among the TyG index, H. pylori infection, and all-cause mortality remains underexplored and warrants further investigation.

Hence, this study aims to explore the relationship between the TyG index and H. pylori infection and investigate whether the associations between the TyG index exposure and all-cause mortality are mediated by H. pylori infection using the data in the NHANES database.

## Materials and methods

### Study design and participants

The NHANES initiative employs a refined and intricate methodology to periodically select a representative sample of the U.S. population. Its primary objective involves evaluating the health and nutritional status of individuals in the United States [14]. To uphold ethical standards, the survey has garnered approval from The National Center for Health Statistics Institutional Review Board. Furthermore, before their inclusion in the study, all participants willingly provided written informed consent. NHANES encompasses a broad spectrum of data, including demographics, dietary patterns, medical examination results, laboratory findings, and responses to questionnaires [15].

**Fig. 1:**
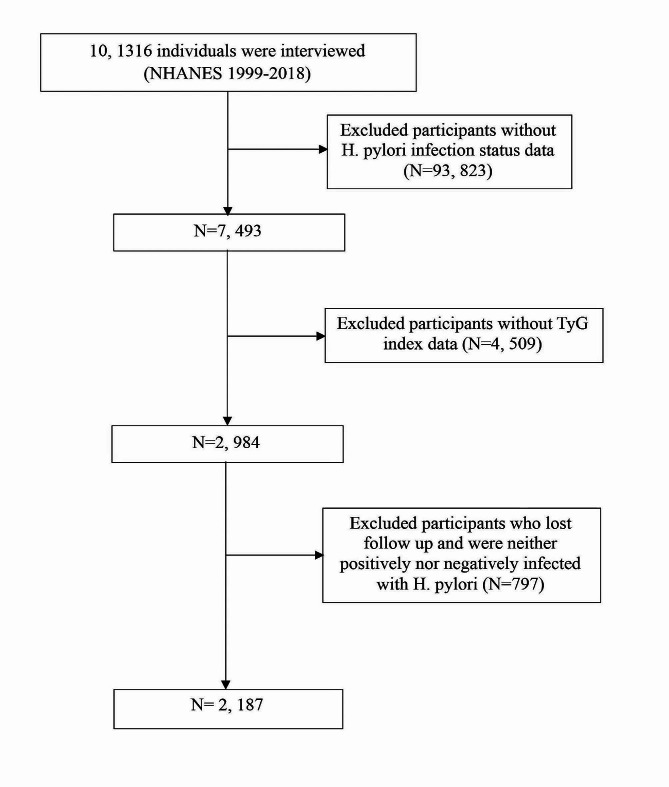
Flow chart for inclusion and exclusion of the study population

Throughout the NHANES 1999–2018 cycle, the study encompassed 101,136 participants. Following the exclusion of individuals lacking H. pylori infection status or the TyG index data, as well as those who were lost to follow-up or did not meet the criteria for H. pylori infection, the remaining subset formed the basis for analysis (Fig. 1).

### TyG index

In this study, the Triglyceride-Glucose (TyG) index is calculated using the formula: TyG index = Ln (TG [mg/dL] × fasting glucose [mg/dL]/2), where Ln represents the natural logarithm, TG is triglycerides, and fasting glucose is measured in mg/dL [16].

### Helicobacter pylori status

H. pylori immunoglobulin G (IgG) antibodies were identified using an enzyme-linked immunosorbent assay (ELISA) kit produced by Wampole Laboratories (Cranbury, NJ) to quantify the levels of IgG antibodies against H. pylori [17]. The participants were categorized into two groups: Hp positive (optical density (OD) value ≥ 1.1) and Hp negative (OD value < 0.9), based on the established ELISA cut-off value. Equivocal results falling within the range of 0.9 to 1.1 were excluded from the analysis to ensure precise statistical outcomes in this study [18].

### Follow-up and endpoint

The mortality status and cause of death for participants were ascertained by cross-referencing their records with the publicly accessible National Death Index files, up until December 31, 2019 (https://www.cdc.gov/nchs/data-linkage/mortality.htm). The median follow-up time for H. pylori-positive individuals was 235 months (interquartile range 185, 243), while for H. pylori-negative patients, it stood at 237 months (interquartile range 230, 243).

### Covariate

Several clinical data, recognized as covariates, were included because of their potential influence on the relationship between the TyG index and H. pylori infection, including age, sex, race, poverty, education, smoking habits, alcohol drinking, diabetes mellitus, hypertension status, cardiovascular disease, blood lipids, and glucose. Hypertension was defined as self-reported hypertension, a systolic blood pressure (SBP) of ≥ 140 mmHg, a diastolic blood pressure (DBP) of ≥ 90 mmHg, or the use of antihypertensive medications [19]. Smoking status was classified into three groups: former, never, and now. Former smokers were defined as those who had previously smoked at least 100 cigarettes but were not presently smoking. Never-smokers included individuals who had either never smoked or had smoked fewer than 100 cigarettes in their lifetime. Now-smokers were participants who had smoked at least 100 cigarettes in their lifetime and reported consuming a non-zero number of cigarettes per day within the past 30 days [20]. Alcohol drinking status was classified into four distinct categories, reflecting their alcohol consumption patterns: Never drinkers (lifetime abstainers), former drinkers (abstinent within the past year), moderate drinkers (1 or 2 drinks per day for females/males, respectively), and heavy drinkers (> 1 or > 2 drinks per day for females/males, respectively, and/or frequent binge drinking) [21, 22]. Moreover, the educational level was categorized into three groups: less than high school, high school, and more than high school. The estimated Glomerular Filtration Rate (eGFR) was determined using the Chronic Kidney Disease Epidemiology Collaboration Equation (CKD-EPI) [23].

### Statistical analysis

The baseline characteristics of participants were summarized and compared between H. pylori-infected and uninfected patients. Continuous variables were expressed as mean (± SD) and compared using either a t-test or Wilcoxon rank-sum test, based on the outcome of the Kolmogorov–Smirnov normality test. Categorical variables were presented as frequency (percentage) and compared using the Chi-square test.

Univariable-adjusted logistic regression analyses were applied to determine the odds ratio (OR) alongside a 95% confidence interval (CI) for assessing the relationship between the TyG index and H. pylori infection. Additionally, the potential nonlinear connections between the TyG index and all-cause mortality were explored using restricted cubic spline (RCS) curves. These curves were positioned at specific percentiles (5%, 35%, 65%, and 95%) within the TyG index distribution [24]. Examining the association of the TyG index with all-cause mortality, cox proportional hazard models were utilized to compute hazard ratios (HRs) and their corresponding 95% CIs. To avoid over-adjustment and optimize data utilization for variables related to the TyG index and mortality, three models were developed: model 1 adjusted for age, sex, BMI, and poverty; model 2 included adjustments for age, sex, BMI, poverty, races, education, smoke, alcohol consumption, hypertension, and waist; and model 3 encompassed uric acid, eGFR, and LDL-C in addition to the adjustments in model 2. Categorizing the TyG index into 4 groups, cox proportional hazards models were implemented, using group 1, Q1, as the reference. The event-free survival rates among these groups were estimated using the Kaplan-Meier method and compared through the log-rank test.

A two-sided *P* < 0.05 was considered statistically significant. All analyses were performed using SPSS version 26.0 (IBM Corp, Armonk, NY) and R (version 4.3.2) [25, 26].

## Results

### Study participants and baseline characteristics

In the final cohort, 2,187 American adults were included, among whom 937 participants tested positive for H. pylori (Fig. 1; Table 1). The average age was 46.39 ± 20.01 years, with males accounting for 47.2% of the sample. In the overall participant group, the mean hemoglobin A1c (HbA1c) level was found to be 5.48 ± 1.03%. In addition, the mean fasting triglyceride level was 143.57 ± 103.11 mg/dL, mean fasting glucose level was 102.47 ± 32.89 mg/dL. More specifically, within the H. pylori-positive group, there was a greater proportion of older individuals, Mexican Americans, individuals with lower socioeconomic status and educational attainment, former drinkers, individuals with diabetes, hypertension, higher body mass index (BMI), elevated fasting triglycerides levels, elevated HbA1c, elevated fasting glucose level.

**Table 1:** Baseline characteristics of participants with different H. pylori infection status

	Overall (*n* = 2187)	Hp negative (*n* = 1250)	Hp positive (*n* = 937)	*P* value
Age (years)	46.39 (20.01)	43.36 (19.88)	50.44 (19.48)	< 0.001
Sex (male %)	1033 (47.2)	567 (45.4)	466 (49.7)	0.047
BMI (kg/m^2^)	27.90 (6.13)	27.73 (6.28)	28.14 (5.93)	0.121
Race (%)				< 0.001
Mexican American	635 (29.0)	226 (18.1)	409 (43.6)	
Non-Hispanic Black	419 (19.2)	203 (16.2)	216 (23.1)	
Non-Hispanic White	926 (42.3)	724 (57.9)	202 (21.6)	
Other Hispanic	148 (6.8)	63 (5.0)	85 (9.1)	
Other Race	59 (2.7)	34 (2.7)	25 (2.7)	
Poverty income ratio	2.56 (1.62)	2.87 (1.64)	2.12 (1.47)	< 0.001
Education (%)				< 0.001
High School	513 (23.5)	347 (27.8)	166 (17.8)	
Less Than High School	844 (38.7)	302 (24.2)	542 (58.2)	
More Than High School	823 (37.8)	599 (48.0)	224 (24.0)	
Wasit (cm)	95.18 (15.49)	94.72 (15.99)	95.81 (14.77)	0.108
Fasting insulin (uU/mL)	13.97 (13.17)	13.30 (10.67)	14.87 (15.86)	0.006
Fasting glucose (mg/dL)	102.47 (34.89)	98.88 (27.32)	107.27 (42.51)	< 0.001
HbA1c (%)	5.48 (1.03)	5.34 (0.81)	5.68 (1.23)	< 0.001
Total bilirubin (umol/L)	9.75 (4.92)	9.77 (5.45)	9.73 (4.11)	0.85
Creatinine (mg/dL)	0.72 (0.45)	0.73 (0.45)	0.72 (0.45)	0.578
Uric acid (mg/dL)	5.32 (1.50)	5.27 (1.47)	5.39 (1.54)	0.077
eGFR	97.80 (24.48)	98.79 (24.62)	96.47 (24.25)	0.029
Fasting total cholesterol (mg/dL)	201.35 (42.33)	199.88 (41.92)	203.31 (42.82)	0.061
Fasting triglyceride(mg/dL)	143.57 (103.11)	135.97 (91.96)	153.72 (115.61)	< 0.001
LDL-C (mg/dL)	122.99 (35.37)	122.18 (35.18)	124.12 (35.62)	0.232
HDL (mg/dL)	50.71 (14.91)	51.64 (14.96)	49.47 (14.75)	0.001
ASCVD = yes (%)	167 (8.6)	81 (7.4)	86 (10.0)	0.057
Smoke (%)				0.079
Former	536 (27.5)	310 (28.5)	226 (26.2)	
Never	1014 (52.0)	574 (52.8)	440 (51.0)	
Now	399 (20.5)	203 (18.7)	196 (22.7)	
Alcohol drink (%)				< 0.001
Former	378 (20.5)	186 (17.9)	192 (23.9)	
Heavy	342 (18.5)	186 (17.9)	156 (19.5)	
Mild	614 (33.3)	377 (36.2)	237 (29.6)	
Moderate	256 (13.9)	166 (15.9)	90 (11.2)	
Never	254 (13.8)	127 (12.2)	127 (15.8)	
Hypertension (%)	809 (37.0)	416 (33.3)	393 (41.9)	< 0.001
SBP (mmHg)	124.77 (20.79)	122.11 (19.10)	128.35 (22.37)	< 0.001
DBP (mmHg)	70.45 (13.79)	70.50 (12.53)	70.40 (15.32)	0.873
Diabetes Mellitus (%)	233 (11.3)	98 (8.4)	135 (15.0)	< 0.001
Stroke = yes (%)	64 (3.3)	31 (2.8)	33 (3.8)	0.284
TyG index	8.70 (0.67)	8.63 (0.63)	8.79 (0.70)	< 0.001

Abbreviation: Helicobacter Pylori (Hp), systolic blood pressure (SBP), diastolic blood pressure (DBP), body mass index (BMI), glycated hemoglobin (HbA1c), estimated glomerular filtration rate (eGFR), high-density lipoprotein (HDL), low-density lipoprotein (LDL-C), atherosclerotic cardiovascular disease (ASCVD), triglyceride-glucose (TyG).

### Associations between TyG index and H. pylori infection

We conducted linear regression analysis to investigate the relationship between variables and H. pylori infection in adults, as outlined in Table S1.

Subsequently, a multivariable logistic regression analysis was conducted to investigate the association between the TyG index and the risk of H. pylori infection, as delineated in Table S2. When assessing the TyG index as a continuous variable, our result suggested that the fourth quartile of the TyG index was strongly correlated with H. pylori infection in the initial Model 0[OR 1.517, 95%CI (1.383,1.664), *P*<0.05]. This correlation persisted even after adjusting for potential confounding factors such as age, sex, BMI, poverty, race, education, smoking, alcohol drinking, hypertension, waist, eGFR, uric acid, and LDL-C in Model 3 [OR 1.189, 95%CI (1.003,1.411), *P*<0.05]

### Correlation between the TyG index exposure and all-cause mortality

**Fig. 2:**
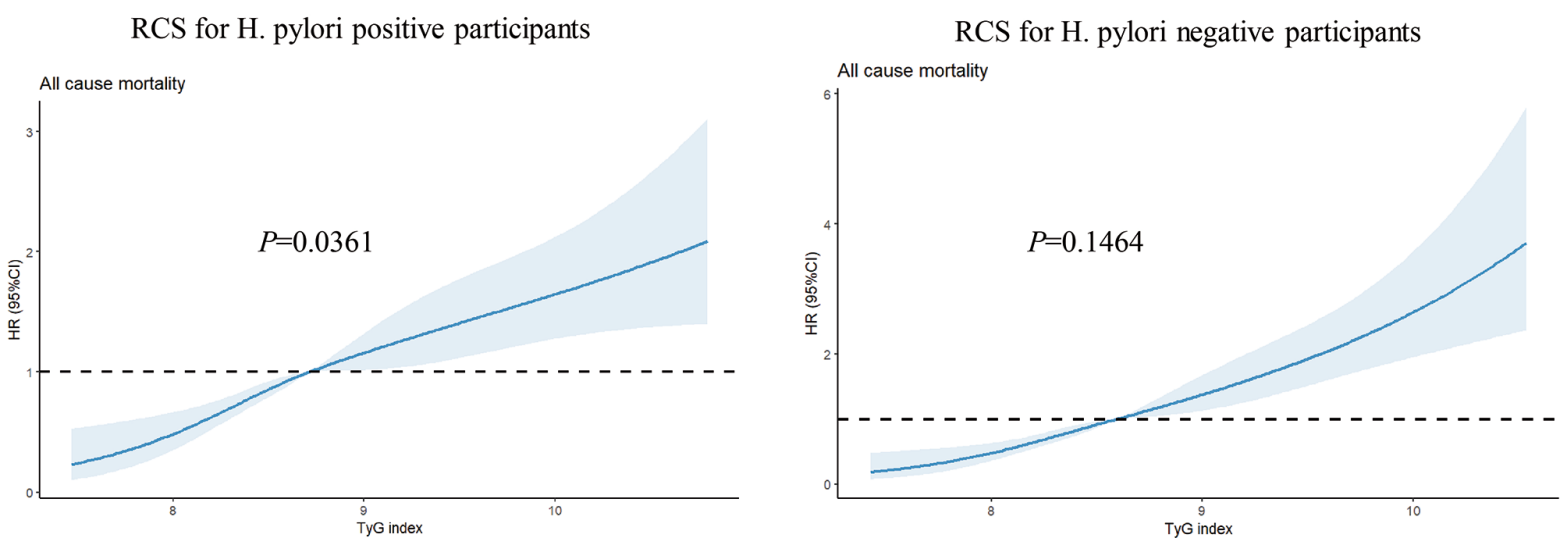
Restricted cubic spline (RCS) for the association between the TyG index and the risks of all-cause death in patients with or without H. pylori infection

Among the total participants, a total of 597 individuals (27.30%) experienced death. The relationship between the TyG index and all-cause mortality was additionally examined through restricted cubic spline (RCS) curves, as illustrated in Fig. 2. The RCS analysis revealed noteworthy findings regarding the linear association between the TyG index, as continuous variables, and the adjusted risk of all-cause mortality in patients with H. pylori infection. The analysis demonstrated non-linear relationships to all-cause mortality in H. pylori-infected patients (*P* = 0.0361). Conversely, in H. pylori-negative patients, the nonlinear relationship did not reach statistical significance (*P* = 0.1464).

**Fig. 3:**
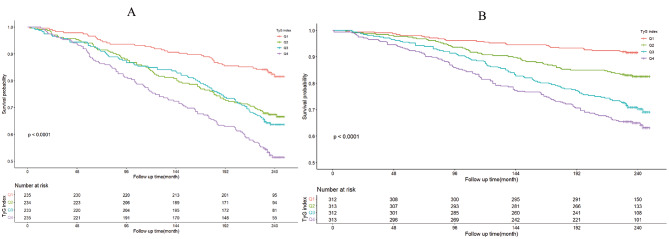
The TyG index association with all-cause mortality. (**A**) Kaplan-Meier survival estimates for all-cause mortality in H. pylori positively infected participants; (**B**) Kaplan-Meier survival estimates for all-cause mortality in H. pylori negatively infected participants

The Kaplan-Meier survival curve analysis demonstrated a significant association between the TyG index and all-cause mortality in H. pylori positively infected participants (Fig. 3), and this correlation persisted after accounting for all potential confounders (HR1.364, 95%CI (1.032,1.804), *P*<0.05). Furthermore, within the subset of patients who tested positive for H. pylori infection, we observed a noteworthy rise in all-cause mortality within the Q4 group when compared to the Q1 group across models M0, M1, M2, and M3 (Table S3). However, in H. pylori negatively infected participants, the Kaplan-Meier survival curve analysis failed to yield a significant association between the TyG index and all-cause mortality after fully adjusting confounding factors. Moreover, among patients who tested negative for H. pylori infection, a significant increase in all-cause mortality was observed solely within the Q4 group when compared to the Q1 group in model M0. However, when considering models M1, M2, and M3, which accounted for potential confounding factors, we did not identify a significant increase in all-cause mortality within the Q4 group when compared to the Q1 group. (Table S4).

## Discussion

This study, through the analysis of cross-sectional data in the NHANES database, found a significant positive correlation between the TyG index and the increased risk of H. pylori infection. This correlation still exists even after adjusting for confounding factors including age, sex, BMI, poverty, race, education, smoking, alcohol drinking, hypertension, waist circumference, eGFR, uric acid, and LDL-C. Moreover, using Kaplan-Meier survival curve analysis to analyze the correlation between the TyG index and all-cause mortality, it was found that in patients with positive H. pylori infection, there is a significant nonlinear correlation between the TyG index and all-cause mortality, and this correlation still exists after adjusting for related confounding factors. However, in patients with negative H. pylori infection, no significant nonlinear correlation was observed between the TyG index and all-cause mortality after adjusting for possible confounding factors. Previous research indicated a significant association between H. pylori infection and the risk of diabetes, particularly type 2 diabetes. A meta-analysis encompassing 41 studies with 9559 participants (4327 cases and 5232 controls) revealed a pooled estimate of the association between H. pylori infection and diabetes at an odds ratio (OR) of 1.27 (95% CI 1.11 to 1.45, *P* = 0.0001, I² = 86.6%). This study found a stronger association with type 2 diabetes compared to type 1 diabetes or diabetes mellitus in general, suggesting that H. pylori infection might be more closely related to insulin resistance-related diseases [27]. Coincidentally, a meta-analysis studying the effect of H. pylori eradication on lipid levels found that H. pylori infection is positively associated with cardiovascular diseases. This study reviewed 24 studies (including four RCTs and 20 non-RCTs) with 5270 participants and found an increase in high-density lipoprotein cholesterol (HDL-C) and triglycerides (TG), but little to no difference in low-density lipoprotein-cholesterol levels [28]. These studies suggested that H. pylori infection might impact glucolipid metabolism, potentially influencing cardiovascular risk and mortality. Our study identified a significant correlation between H. pylori infection and the TyG index. Elevated TyG index levels were linked to greater insulin resistance and an elevated risk of cardiovascular complications [29, 30]. Consequently, individuals with higher TyG index values might face an increased risk of mortality [12]. Simultaneously, our research also revealed that the TyG index was positively correlated with all-cause mortality in patients with H. pylori infection, suggesting a significant link between metabolic dysregulation and mortality outcomes in this patient group. This finding not only confirms the results of previous research but also expands upon them by providing additional insights into the relationship between HP infection and metabolic indicators.

In addition to H. pylori infection increasing the risk of metabolic disorders, metabolic abnormalities such as diabetes may also increase the risk of H. pylori infection. For instance, diabetic individuals often have a compromised immune system, making them more susceptible to infections, including H. pylori. Furthermore, changes in the gastric mucosa associated with diabetes may create a more favorable environment for H. pylori colonization [31]. In the study conducted by Chen et al., a significant positive nonlinear relationship was found between HbA1c and H. pylori infection. This study also suggested long-term H. pylori infection increased HbA1c levels, while H. pylori eradication might decrease HbA1c levels [32]. These findings demonstrate the complexity of the relationship between H. pylori infection and metabolic disorders, suggesting the need for more research focused on the correlation between H. pylori infection and abnormalities in glucose and lipid metabolism.

To the best of our knowledge, our study represents the initial investigation into the association between the TyG index and H. pylori infection, offering valuable insights to augment the existing body of knowledge. This research has the potential to refine risk stratification and guide personalized approaches to healthcare for individuals with H. pylori infection and further drug targets, ultimately contributing to improved patient outcomes and enhanced geriatric care. In light of recent findings, it becomes evident that H. pylori exerts a significant influence beyond the gastrointestinal tract, playing a pivotal role in metabolism and directly impacting mortality. This revelation holds profound implications for future research directions and underscores the critical importance of HP eradication. The accumulation of supporting evidence further emphasizes the urgency in addressing H. pylori eradication, heightening the concerns in this domain.

## Limitations

Firstly, the observational nature of our study design precludes the inference of causality. Confirmatory evidence is warranted through prospective studies with larger sample sizes. Secondly, participants in NHANES may not be fully representative of the global population, and the applicability of our findings to different ethnicities remains uncertain.

## Conclusion

In our sample, a nonlinear relationship has been identified between the TyG index and H. pylori infection within the US population. Elevated levels of the TyG index suggest a heightened risk of H. pylori infection among US adults. Furthermore, the TyG index exhibits a positive association with all-cause mortality in H. pylori-positive participants.

### Electronic supplementary material

Below is the link to the electronic supplementary material.

Supplementary Material 1

## Data Availability

No datasets were generated or analysed during the current study. The raw data supporting the conclusions of this article can be found here: https://www.cdc.gov/nchs/nhanes/.
