# Supplementary material for: Association of triglyceride-glucose index with helicobacter pylori infection and mortality among the US population
Source: Diabetol Metab Syndr. 2024 Aug 1;16:187. doi: 10.1186/s13098-024-01422-9 (PMC11293276; doi:10.1186/s13098-024-01422-9)
Supplement: Supplementary file 1 — Supplementary Material 1 [file 13098_2024_1422_MOESM1_ESM.docx]

Table S1 Risk factors for H. pylori infection in adults in NHANES 1999-2018

| Variables | β | Standard Error | *P* value | OR | 95% CI |
| --- | --- | --- | --- | --- | --- |
| Age | 0.01 | 0.00 | <0.001 | 0.01 | (0.01 ~ 0.01) |
| Sex | 0.04 | 0.02 | 0.043 | 0.04 | (0.01 ~ 0.08) |
| Race | -0.30 | 0.02 | <0.001 | -0.30 | (-0.35 ~ -0.26) |
| Education | 0.14 | 0.02 | <0.001 | 0.14 | (0.09 ~ 0.19) |
| BMI | 0.00 | 0.00 | 0.121 | 0.00 | (-0.00 ~ 0.01) |
| Poverty | -0.07 | 0.01 | <0.001 | -0.07 | (-0.08 ~ -0.06) |
| Diabetes mellitus | -0.17 | 0.03 | <0.001 | -0.17 | (-0.24 ~ -0.10) |
| Waist | 0.00 | 0.00 | 0.108 | 0.00 | (-0.00 ~ 0.00) |
| ASCVD | 0.08 | 0.04 | 0.047 | 0.08 | (0.01 ~ 0.16) |
| Stroke | 0.08 | 0.06 | 0.230 | 0.08 | (-0.05 ~ 0.20) |
| Hypertension | 0.09 | 0.02 | <0.001 | 0.09 | (0.05 ~ 0.13) |
| Smoke | 0.04 | 0.02 | 0.062 | 0.04 | (-0.00 ~ 0.08) |
| Alcohol drink | -0.07 | 0.02 | 0.002 | -0.07 | (-0.11 ~ -0.03) |
| Total bilirubin | -0.00 | 0.00 | 0.850 | -0.00 | (-0.00 ~ 0.00) |
| Creatinine | -0.01 | 0.02 | 0.578 | -0.01 | (-0.06 ~ 0.03) |
| Uric acid | 0.01 | 0.01 | 0.077 | 0.01 | (-0.00 ~ 0.03) |
| eGFR | -0.01 | 0.00 | 0.029 | -0.01 | (-0.99 ~ -0.01) |
| LDL-C | 0.00 | 0.00 | 0.232 | 0.00 | (-0.00 ~ 0.00) |
| TyG index | 0.09 | 0.02 | <0.001 | 0.09 | (0.06 ~ 0.12) |

Table S2 Associations between the TyG index and H. pylori infection.

| Variables |  |  | | OR (95%CI) | |  | |
| --- | --- | --- | --- | --- | --- | --- | --- |
| Continuous variables  TyG index | Model 0  1.517(1.383,1.664) *** | | Model 1^1^  1.117(0.995,1.255) | | Model 2^2^  1.155(1.016,1.313) * | | Model 3^3^  1.189(1.003,1.411) * |
| Categorical variable |  | |  | |  | |  |
| Q1 group | Ref | | Ref | | Ref | | Ref |
| Q2 group | 1.257(1.039,1.520) * | | 0.874(0.701,1.090) | | 0.835(0.646,1.077) | | 0.797(0.607,1.045) |
| Q3 group | 1.372(1.134,1.661) *** | | 0.823(0.656,1.032) | | 0.850(0.654,1.105) | | 0.790(0.597,1.045) |
| Q4 group | 1.925(1.604,2.312) *** | | 1.048(0.833,1.319) | | 1.078(0.822,1.413) | | 0.978(0.730,1.311) |

^a^ Model 1 adjusted for age, sex, BMI, poverty

^b^ Model 2 adjusted for age, sex, BMI, poverty, races, education, smoke, alcohol drink, hypertension and waist.

^c^ Model 3 adjusted for age, sex, BMI, poverty, races, education, smoke, alcohol drink, hypertension, waist, eGFR, uric acid, and LDL-C.

**P < 0.05, **P < 0.01, ***P < 0.001*.

Table S3 Associations of the TyG index and all-cause mortality in H. pylori positively infected participants.

| Variables |  |  | | HR (95%CI) | |  | |
| --- | --- | --- | --- | --- | --- | --- | --- |
| Continuous variables  TyG index | Model 0  1.661(1.453,1.900) *** | | Model 1^1^  1.198(1.005,1.427) * | | Model 2^2^  1.134(0.923,1.393) | | Model 3^3^  1.364(1.032,1.804) * |
| Categorical variable |  | |  | |  | |  |
| Q1 group | Ref | | Ref | | Ref | | Ref |
| Q2 group | 2.080(1.424,3.039) *** | | 1.173(0.754,1.827) | | 1.264(0.785,2.037) | | 1.313(0.786,2.194) |
| Q3 group | 2.285(1.573,3.319) *** | | 1.121(0.729,1.722) | | 1.201(0.745,1.937) | | 1.129(0.670,1.903) |
| Q4 group | 3.355(2.345,4.799) *** | | 1.575(1.036,2.394) * | | 1.652(1.018,2.680) * | | 1.696(1.003,2.866) * |

^a^ Model 1 adjusted for age, sex, BMI, poverty

^b^ Model 2 adjusted for age, sex, BMI, poverty, races, education, smoke, alcohol drink, hypertension and waist.

^c^ Model 3 adjusted for age, sex, BMI, poverty, races, education, smoke, alcohol drink, hypertension, waist, eGFR, uric acid, and LDL-C.

**P < 0.05, **P < 0.01, ***P < 0.001*.

Table S4 Associations of the TyG index and all-cause mortality in H. pylori negatively infected participants.

| Variables |  |  | | HR (95%CI) | |  | |
| --- | --- | --- | --- | --- | --- | --- | --- |
| Continuous variables  TyG index | Model 0  2.272(1.931,2.674) *** | | Model 1^1^  2.272(1.931,2.674) * | | Model 2^2^  2.272(1.931,2.674) | | Model 3^3^  0.922(0.660,1.289) |
| Categorical variable |  | |  | |  | |  |
| Q1 group | Ref | | Ref | | Ref | | Ref |
| Q2 group | 2.172(1.360,3.468) *** | | 0.640(0.375,1.094) | | 0.589(0.328,1.057) | | 0.564(0.311,1.021) |
| Q3 group | 3.946(2.553,6.099) *** | | 0.825(0.495,1.375) | | 0.734(0.420,1.280) | | 0.715(0.401,1.277) |
| Q4 group | 5.020(3.275,7.695) *** | | 0.896(0.539,1.489) | | 0.766(0.436,1.348) | | 0.614(0.336,1.122) |

^a^ Model 1 adjusted for age, sex, BMI, poverty

^b^ Model 2 adjusted for age, sex, BMI, poverty, races, education, smoke, alcohol drink, hypertension and waist.

^c^ Model 3 adjusted for age, sex, BMI, poverty, races, education, smoke, alcohol drink, hypertension, waist, eGFR, uric acid, and LDL-C.

**P < 0.05, **P < 0.01, ***P < 0.001*.
